# JBO 2020 List of Reviewers

**DOI:** 10.1117/1.JBO.26.1.010102

**Published:** 2021-01-19

## Abstract

List of reviewers who served JBO in 2020.

The *Journal of Biomedical Optics* sincerely thanks the following individuals who served as reviewers in 2020. The success of our publication hinges on the voluntary contributions of time and energy put forth by these professionals.

Steven Adie
Osman Ahsen
Yoshihisa Aizu
Alba Alfonso-Garcia
Thomas Allen
Clemens Alt
Robert Amelard
Arjen Amelink
Marco Andreana
Andreas Andreou
Mohamed Ansari
Alexander Antaris
Brian Applegate
Praveen Arany
Hidenobu Arimoto
Muhammad Ramish Ashraf
Yasuhiro Awatsuji
Marina Bakaric
Wesley Baker
Yijun Bao
Timothy Baran
Utku Baran
Randall Barbour
Jennifer Barton
Alexey Bashkatov
Adam Bauer
Paul Beard
Andrey Belikov
Brian Beliveau
Muyinatu Bell
Fouzi Benboujja
Renzhe Bi
Walter Blondel
Albert Boccara
Sarah Bohndiek
Enrico Borrelli
Nada Boustany
Audrey Bowden
Ralf Brinkmann
J. Quincy Brown
Michael Brown
Yang Bu
Maria Burdanova
Hugh Byrne
Lucas Cahill
Xu Cao
Stefan Carp
Jeffrey Cassidy
Jonathan Celli
Andrew Chalmers
Mason Tianyi Chen
Nanguang Chen
Peng Chen
Shichao Chen
Shihchi Chen
Olga Cherkasova
Regine Choe
Woo Choi
Bonghwan Chon
Shau Poh Chong
Zhongdi Chu
Zachary Coker
Olga Conde
Christian Crouzet
Arnaud Cuisset
Andrea Curatolo
Michael Daly
Arash Darafsheh
Rupsa Datta
Bin Deng
Damon DePaoli
Michele Desjardins
Michele Diana
U. S Dinish
Ana Doblas
Jianfei Dong
James Dormer
Alexander Dumont
Adam Eggebrecht
Jonathan Elliott
Katherine Ember
Stanislav Emelianov
Thomas Ertl
Qiyin Fang
Felix Fanjul-Velez
Michael Fanous
Sergio Fantini
Peng Fei
Marc Feldman
Jinchao Feng
Ting Feng
Farzad Fereidouni
Raul Fernandez Rojas
Fabio Feroldi
Reto Fiolka
Martin Fischer
Jorge Flores Moreno
Martin Frenz
Nathaniel Fried
Ekaterina Galanzha
Liang Gao
Jerome Gateau
Irene Georgakoudi
Arkadiusz Gertych
Yilbert Gimenez
Martina Giovannella
Natalia Gladkova
Adam Glaser
Randolph Glickman
Anuradha Godavarty
Matthew Goodwin
Dimitris Gorpas
Jason Granstedt
Andree-Anne Grosset
Ignacy Gryczynski
Eugenio Gutierrez
Rainer Haak
Rachael Hachadorian
Steffen Hackbarth
Michael Hamblin
Omnia Hamdy
Daniel Hammer
Songfeng Han
Hideaki Haneishi
Sveinn Hardarson
Andrew R. Harvey
Carole Hayakawa
Honghui He
Shenghua He
Sicong He
Maria del Socorro Hernandez-Montes
Brian Hill
Harish Hiriyannaiah
Jon Holmes
Song Hu
Gereon Huettmann
Brady Hunt
Tiancheng Huo
Theodore Huppert
Nicusor Iftimia
Ilko Ilev
Minbiao Ji
Mengyu Jia
Renu John
Jesse V. Jokerst
James Joseph
Agnieszka Kaczor
Takashi Kakue
Francis Kalloor Joseph
Tschackad Kamali
Mikhail Kandel
Jeon Woong Kang
Stephen Kanick
Karol Karnowski
Kavon Karrobi
Deepa Kasaragod
Bjoern Kemper
Brendan Kennedy
Asifullah Khan
Davood Khodadad
Alwin Kienle
Changseok Kim
Chulhong Kim
Alex Ko
Tiffany Ko
Lisa Kobayashi
Anne Koenig
Michael Kolios
Gennady Komandin
Sergey Komarov
Can Koyuncu
Peter Kronenberg
Konstantin Kudrin
Roman Kuranov
Daria Kuznetsova
Lily Lai
Guy Lamouche
Pierre Lane
Kirill Larin
Marcel Lauterbach
Ekaterina Lazareva
V. N. Du Le
Frederic Leblond
Chau-Hwang Lee
Hsiang-Chieh Lee
John Lee
Frederic Lesage
Richard Levenson
Chenxi Li
Chunqiang Li
Dawei Li
Hui Li
Jiao Li
Jiasong Li
Lei Li
Li Li
Lin Li
Mucong Li
Qingli Li
Shuying Li
Ting Li
Antonia Lichtenegger
Adam Liebert
Lothar Lilge
Weichiang Lin
Changgeng Liu
Gangjun Liu
Jungping Liu
Linbo Liu
Quan Liu
Yang Liu
Guolan Lu
Andrei Lugovtsov
Wangxi Luo
Fiona Lyng
Cheng Ma
Steen Madsen
Boris Majaron
Srivalleesha Mallidi
Zbignevs Marcinkevics
Laura Marcu
Zachary Markow
Mikael Marois
Dominik Marti
Mohammad Mehrmohammadi
Zhaokai Meng
Andrew Metha
Ilya Mikhelson
Miguel Mireles-Nunez
Shachi Mittal
Andreia Moco
Suman Mondal
Inkyu Moon
Ali-Reza Moradi
Joshua Morgan
Stephen Morgan
Moein Mozaffarzadeh
Mircea Mujat
Oleg Nadyarnykh
Sreyankar Nandy
Pavel Nikitin
Meir Nitzan
Santi Nonell
Ioan Notingher
Thomas O’Sullivan
Paul Okagbare
Amy Oldenburg
Luis Oliveira
Mauricio Ortiz-Gutierrez
Asael Papour
Seonyeong Park
YongKeun Park
Yannis Paulus
Ivan Pelivanov
Leilei Peng
Rozhin Penjweini
Vijitha Periyasamy
Daqing Piao
Mark Pierce
Arthur Plante
Alexey Popov
Eric Potma
Manojit Pramanik
Guillem Pratx
G. L. Puyo
Le Qiu
Xiangyu Quan
Timothy Quang
Raksha Raghunathan
Narasimhan Rajaram
Rajagopal Ramasubramaniam
Jessica Ramella-Roman
Robert Raphael
Christopher Raub
Aniruddha Ray
Rebecca Richards-Kortum
Yair Rivenson
Andres Rodriguez
Jeremy Rogers
Peter Rolfe
Vincent Rossi
Francesca Rossi Paoletti
Luigi Rovati
Sandra Rugonyi
Alberto Ruiz
Rolf Saager
Yusuke Saita
Sava Sakadzic
Inga Saknite
Felix Scholkmann
Peter Schunemann
Dietrich Schweitzer
Ikbal Sencan
Babak Shadgan
Natan Shaked
Yu Shang
Benjamin Sherlock
Weichuan Shih
Alexander Shkurinov
Michael Shribak
Xiao Shu
Mira Sibai
Ronald Silverman
Kanwarpal Singh
Mayanglambam Singh
Melissa Skala
Eric Snider
Sergei Sokolovski
Janis Spigulis
Vishal Srivastava
Ronald Sroka
Wiendelt Steenbergen
Samuel Streeter
Ulas Sunar
Kung-Bin Sung
Mahesh Swami
Syeda Tabassum
Masanori Takabayashi
Jianbo Tang
Suresh Thennadil
Robert Thomas
Greg Thurber
Jerilyn Timlin
Snow Tseng
Vassiliy Tsytsarev
Labrinus van Manen
Rudolf Verdaasdonk
Phuong Vincent
I. Alex Vitkin
William Vogt
Sunil Vyas
Dushan Wadduwage
Vincent Patrick Wallace
Alex Walsh
Hao Wang
Hui Wang
Ping Wang
Quan Wang
Xiaolei Wang
Xueding Wang
Wataru Watanabe
Dale Waterhouse
Xunbin Wei
Brian White
David Wilson
Jesse Wilson
Maciej Wojtkowski
Terence Wong
Binlin Wu
Yicong Wu
Jun Xia
Peng Xia
Wenfeng Xia
Chen Xu
Guan Xu
Ronald Xu
Egor Yakovlev
Phaneendra Yalavarthy
Bin Yang
Jiamiao Yang
Xusan Yang
Gang Yao
Junjie Yao
Xincheng Yao
Xinwen Yao
Mohammad Yaseen
Lawrence Yip
Arjun Yodh
Bing Yu
Jie Yuan
Vladimir Zaitsev
Mark Zarella
Cristina Zavaleta
Haishan Zeng
Jin Zhang
Wei Zhang
Xiaohui Zhang
Yide Zhang
Zhiqing Zhang
Hubin Zhao
Xiangwei Zhao
Hao Zhou
Jiaqi Zhu
Liren Zhu
Shouping Zhu
Fernando Zvietcovich

